# Improving Toxicity Assessment of Pesticide Mixtures: The Use of Polar Passive Sampling Devices Extracts in Microalgae Toxicity Tests

**DOI:** 10.3389/fmicb.2016.01388

**Published:** 2016-09-09

**Authors:** Sandra Kim Tiam, Vincent Fauvelle, Soizic Morin, Nicolas Mazzella

**Affiliations:** Institut National de Recherche en Sciences et Technologies pour l'Environnement et l'AgricultureUR EABX, Cestas, France

**Keywords:** POCIS, Chemcatcher, passive samplers, biofilms, low dose, PICT, environmental risk assessment

## Abstract

Complexity of contaminants exposure needs to be taking in account for an appropriate evaluation of risks related to mixtures of pesticides released in the ecosystems. Toxicity assessment of such mixtures can be made through a variety of toxicity tests reflecting different level of biological complexity. This paper reviews the recent developments of passive sampling techniques for polar compounds, especially Polar Organic Chemical Integrative Samplers (POCIS) and Chemcatcher® and the principal assessment techniques using microalgae in laboratory experiments. The progresses permitted by the coupled use of such passive samplers and ecotoxicology testing as well as their limitations are presented. Case studies combining passive sampling devices (PSD) extracts and toxicity assessment toward microorganisms at different biological scales from single organisms to communities level are presented. These case studies, respectively, aimed (i) at characterizing the “toxic potential” of waters using dose-response curves, and (ii) at performing microcosm experiments with increased environmental realism in the toxicant exposure in term of cocktail composition and concentration. Finally perspectives and limitations of such approaches for future applications in the area of environmental risk assessment are discussed.

## Introduction

Aquatic organisms are exposed to a large variety of natural and anthropogenic stressors. Environmental conditions such as temperature, nutrients, light, flow, are ever changing, as well as chemical contaminants. Rivers are contaminated by complex mixtures of organic and inorganic substances, generally present at low concentrations. Risk assessment thus requires consider this complexity in exposure conditions (Chèvre and Gregorio, 2013).

Recent developments of passive sampling techniques allowed, (i) to better consider the diversity of co-occurring substances in the environment, (ii) to lower their detection limits, and (iii) to focus on the freely dissolved fraction instead of the total amount of contaminants, which is more comparable to the *in situ* bioavailability. These advantages of passive sampling compared to spot sampling relied on the capacity of the device to accumulate contaminants over a defined period of time. They thus provide a more complete panorama of the contamination. Among passive sampling devices (PSDs), Polar Organic Chemical Integrative Samplers (POCIS) and Chemcatchers® are used to quantify time-weighted-average concentrations of organic substances in aquatic environments, especially for hydrophilic pesticides (see Alvarez et al., 2004). Depending on their configuration, both samplers are able to accumulate linearly dissolved polar contaminants over a period of time. Then, complex mixtures of polar organic compounds (e.g., pesticides, pharmaceuticals) can be extracted from these passive samplers to perform toxicity tests at different biological scales from single organisms to community levels.

Societal demand calls for increasing ecological relevance of ecotoxicological testing, in particular regarding the biological endpoints used (Artigas et al., 2012). Biofilms have been used in ecotoxicology because of their high degree of environmental relevance (Sabater et al., 2007). This community (composed of microalgae, bacteria, fungi, protozoa, etc.) integrates a higher amount of biological complexity than standardized tests using single model species or sub-cellular endpoints conventionally used in toxicity testing. Biofilm-based ecotoxicology increases the predictive power of impacts at the ecosystem scale, compared to the extrapolation of results disconnected from the natural complexity (diversity of organisms and of their relations). These communities are used successfully in ecotoxicology, in the laboratory or *in situ*, to assess impacts of diverse contaminants (e.g., Guasch et al., 2012). Laboratory experiments with biofilms involve single substances or simple (binary, ternary) combinations. However, the determination of the ecotoxicity of complex mixtures of toxicants could hardly be achieved this way, given the multiplicity of possible cocktails (in terms of composition, and concentrations), preventing to test all the combinations likely to be found *in situ*.

### I. passive sampling

Evaluation of water quality relative to chemical substances concentration is generally based on spot sampling (2000/60/EC). This type of sampling is quick, easy to realize and relatively low cost but the data obtained are limited regarding temporal representativeness and detection limits. For example, in the case of peaks of contaminants occurring within hours, spot sampling will not offer a good picture of water contamination. Limitations of spot sampling strategies also concern the detection limit of compounds present under the ng/L. An interesting option in order to compensate for these limitations can be found in passive sampling.

#### History of PSDs development

History of passive sampling starts early 70's with the development of devices able to accumulate atmospheric pollutants by diffusion (Palmes and Gunnison, 1973) or permeation (Reiszner and West, 1973). Two decades by then, the first passive samplers adapted for aquatic environments appear with the SPMD (Semipermeable Membrane Device; Huckins et al., 1990) used for sampling of non-polar organic compounds and then the DGT (Diffusive Gradient in Thin-film; Davison and Zhang, 1994) specific for inorganic contaminants such as metals and phosphates. By then, a considerable number of passive samplers have been developed for medium polar contaminants: MESCO (Membrane-Enclosed Silicone Collector; Paschke et al., 2006), LDPE (Low-Density Polyethylene; Müller et al., 2001), SR (Silicone Rubber; Rusina et al., 2010), Chemcatcher® (Kingston et al., 2000), POCIS (Polar Organic Chemical Integrative Sampler; Alvarez et al., 2004). Several recent developments reported the adaptation of POCIS and DGT for the sampling of highly polar compounds such as antibiotics (e.g., sulfamethoxazole), perfluorinated chemicals, and pesticides (e.g., glyphosate, 2,4-D) (Fauvelle et al., 2012, 2015; Chen et al., 2013; Kaserzon et al., 2014a).

#### Principles of passive sampling

The general principles of passive sampling have been reviewed exhaustively by Vrana et al. (2005). Briefly, PSDs can be compared to a standardized compartment introduced in the water column (or other media such as sediment, soil, etc.), able to accumulate the contaminants of interest. The flux of contaminants from water to the sample is theoretically governed by chemical diffusion, and is assumed to follow a first-order kinetic composed of (i) an initial integrative phase, and (ii) a later equilibrium or saturation of the receiving phase. During the integrative phase, the receiving phase of the sampler is considered as an infinite sink, ensuring a difference of chemical activity between both the water and the sampler receiving phase; and the time weighted averaged concentration *C*_*w*_ in water (ng/L) can be deduced from *N*_*s*_ the amount of analytes measured in the sampler (ng) as presented in the equation:

(1) $$\begin{array}{c} C_{W}=\frac{N_{s}}{R_{s}\times t} \end{array}$$

where *R*_*s*_ the sampling rate constant of the analyte from the water to the sampler (L/d), and *t* the exposure duration (days). Obviously, the back calculation of *C*_*w*_ implies the prior determination of *R*_*s*_ under controlled conditions of *C*_*w*_, temperature, flow rate, etc.

#### Passive sampling: for a better characterization of environmental contaminations

PSDs propose several advantages compared to spot sampling inherent to their accumulation properties. First, they give a more realistic representation of environmental contamination by integrating the contamination during a determinate period of time while spot sampling only gives a picture of the contamination at the precise sampling time. This characteristic is critical considering environments where contaminations are transiting, like in rivers where the duration of the contamination peaks can be measured in hours. Indeed the analysis of the compounds extracted from a passive sampler exposed in the field will give a better estimation of water contamination during this period than one analysis performed on grab water sampled one time during the typical 2 weeks passive sampler exposure. Second, passive sampling allows a lowering of detection limits since *in situ* preconcentration rates (tenth of a liter to several liters per day of exposure) are much higher than that generally applied during a conventional solid phase extraction procedure for a spot sample (Allan et al., 2006; Harman et al., 2012; Morin et al., 2012a). This gain in detection limit is particularly relevant when considering chemical substances that the environmental quality standards (EQS) values defined by the UE Water framework Directive (2000/60/EC) are below the ng/L threshold [e.g., 0.17 ng/L for benzo(a)pyrene and 0.08 ng/L for the pesticide cypermethrin]. Third, as passive samplers are able to accumulate specifically the free dissolved fraction of the target substances, they are more likely to catch only the bioavailable fraction of contaminants (Harman et al., 2012). In the case of polar substances, such fraction can be achieved by filtration of the sample at a given cutoff (generally from 0.1 to 0.45 μm), although equilibrium between dissolve and particulate phases could be altered during sample treatment and storage steps (Allan et al., 2006). In the case of ionic substances, including both inorganic and organic species (e.g., metals, phosphates, some antibiotics, or ionic pesticides like glyphosate), speciation has also to be taken into account, since complexation tends to inactivate their toxicity (Tsui et al., 2005; Zhou et al., 2013). This paper only reviews the passive samplers which were used for subsequent biotesting (i.e., POCIS and Chemcatcher®). They were chosen because of their wide spectrum and high accumulation capacity. A table summarizes in a non-exhaustive way their performances for the sampling of various classes of polar pesticides (Table 1).

**Table 1:** **Sampling of different polar pesticides by different configurations of POCIS and Chemcatcher**.

**Contaminant class**	**Device configuration**	**Performances**	**References**
Neutral pesticides Phenylureas, chloroacetanilides, triazines	POCIS 45.8 cm^2^ 200 mg Oasis HLB PES 0.1 μm	*t*_1/2_ > 14 d *R_s_* = 37–400 mL d^−1^	Lissalde et al., 2011; Vermeirssen et al., 2012; Morin et al., 2013
	POCIS 45.8 cm^2^ 600 mg Oasis HLB PES 0.1 μm	*t*_1/2_ > 20 d *R_s_* = 466 mL d^−1^ for atrazine	Fauvelle et al., 2014
	POCIS 45.8 cm^2^ 200 mg Oasis MAX PES 0.1 μm	*t*_1/2_ > 21 d (2–6 d for triazine metabolites) *R_s_* = 65–303 mL d^−1^	Li et al., 2011; Fauvelle et al., 2012
	POCIS 16 cm^2^ 600 mg Oasis HLB PES 0.45 μm	*t*_1/2_ > 26 d *R_s_* = 163–298 mL d^−1^	Kaserzon et al., 2014b
	Chemcatcher 17.5 cm^2^ C18 PES 0.2 μm	*t*_1/2_ > 14 d (3 d for carbendazim) *R_s_* = 45–81 mL d^−1^	Camilleri et al., 2012
	Chemcatcher 17.5 cm^2^ SDB-RPS No membrane	*t*_1/2_ ~ 5 d *R_s_* = 260–770 mL d^−1^	Fernández et al., 2014
	Chemcatcher 17.5 cm^2^ SDB-XC No membrane	*t*_1/2_ > 14 d *R_s_* = 120–440 mL d^−1^	Gunold et al., 2008
	Chemcatcher 17.5 cm^2^ SDB-RPS PES 0.2 μm	*t*_1/2_ > 14 d *R_s_* = 56–200 mL d^−1^	Kaserzon et al., 2014b; Moschet et al., 2015
	Chemcatcher 17.5 cm^2^ SDB-RPS PES 0.45 μm	*t*_1/2_ > 26 d *R_s_* = 88–151 mL d^−1^	Kaserzon et al., 2014b
	Chemcatcher 17.5 cm^2^ SDB-XC PES 0.45 μm	*t*_1/2_ = 2–5 d *R_s_* = 306–747 mL d^−1^	Kaserzon et al., 2014b
	Chemcatcher 17.5 cm^2^ SDB-RPS PES 0.1 μm	*t*_1/2_ > 8 d *R_s_* = 32–87 mL d^−1^	Vermeirssen et al., 2012
Acidic herbicides Aryloxyacids, sulfonylurea, ESA and OXA metabolites of chloroacetanilides	POCIS 45.8 cm^2^ 200 mg Oasis HLB PES 0.1 μm	*t*_1/2_ < 7 d (ESA/OA metabolites 10 d) *R_s_* = 37–343 mL d^−1^	Mazzella et al., 2007; Fauvelle et al., 2012; Morin et al., 2013
	POCIS 45.8 cm^2^ 200 mg Oasis HLB Nylon 10 μm	*t*_1/2_ < 4.6 j *R_s_* = 60–136 mL.j^−1^	Belles et al., 2014
	POCIS 16 cm^2^ 600 mg Strata X PES 0.45 μm	*t*_1/2_ = 3, 8–12 d *R_s_* = 168–270 mL d^−1^	Kaserzon et al., 2014b
	POCIS 45.8 cm^2^ 200 mg Oasis MAX PES 0.1 μm	*t*_1/2_ ≥ 14 d *R_s_* = 48–302 mL d^−1^	Fauvelle et al., 2012
	POCIS 3.14 cm^2^ 200 mg Oasis HLB PES 0.1 μm	*t*_1/2_ ≥ 35 d *R_s_* = 2–12 mL d^−1^	Fauvelle et al., 2014
	Chemcatcher 17.5 cm^2^ SDB-RPS PES 0.45 μm	*t*_1/2_ ≥ 14 d *R_s_* = 20–40 mL d^−1^	Camilleri et al., 2012
	Chemcatcher 17.5 cm^2^ SDB-RPS PES 0.1 μm	*t*_1/2_ > 8 d *R_s_* = 21–23 mL d^−1^	Vermeirssen et al., 2012

Device configuration is documented in terms of surface area, sorbent and membrane pore size. t_1/2_, half-time to the equilibrium; R_s_, sample rate; PES, polyethersulfone; SBD-RPS, Empore SDB-RPS disk; SBD-XC, Empore SDB-XC disk.

#### Passive samplers for polar pesticides

##### The polar organic chemical integrative sampler (POCIS)

POCIS has been developed at the beginning of the 2000's because of the lack of passive samplers for polar organic compounds (Alvarez, 1999; Alvarez et al., 2004). It is to date one of the mostly used polar PSD, and some papers review extensively their application (Harman et al., 2012; Morin et al., 2012a). POCISs are made of a microporous sorbent phase sequestered between two membranes of polyethersulfone (PES, usually pores diameter 0.1 μm) and maintained by two stainless steel rings. It exists under two commercial configurations “pesticides” and “pharmaceutical” that differ from the nature of their sorbent. Besides their denomination, ≪ pharmaceutical POCIS ≫ revealed to be more adapted to the sampling of a larger range of polar pesticides, i.e., for compounds having a log *K*_*ow*_ between 1 and 4 (Mazzella et al., 2007). For biotesting applications, performance reference compounds (PRCs) are not used to correct from environmental exposure conditions, since they could induce a high baseline toxicity (Pesce et al., 2011).

##### The Chemcatcher®

Chemcatcher® was first developed for non-polar contaminants (Kingston et al., 2000), and was then adapted for metals (Björklund Persson et al., 2001), organo metallic compounds (Aguilar-Martínez et al., 2011), and a wide range of polar contaminants (Moschet et al., 2015). It was the object of recent and extensive reviews (Charriau et al., 2016; Lissalde et al., 2016). Briefly, its polar configuration consists of a one-side opening housing (exposure area between 14.5 and 17.5 cm^2^ depending on the evoluting design) that includes a Empore disk receiving phase (C18 or SDB-XC or SDB-RPS) and an optional membrane (PES 0.1–0.45 μm pore size). Chemcatcher® thus allows many different combinations, with the additional advantage to have a stationary receiving phase which increase theoretically the reproducibility of the data compared to the POCIS. C18 and SDB-XC (styrenedivinylbenzene exchange) are adapted for the sampling of medium polar compounds (reversed phase sorbent), whereas SDB-RPS (styrenedivinylbenzene reverse phase sulfonate) is used for highly polar compounds (log *K*_*ow*_ down to –2 approximately). Chemcatcher® procedure includes a conditioning step of the Empore disks before exposure (usually methanol and water), which constitutes a practical drawback compared to the POCIS. The addition of a PES membrane induces a decrease of the overall mass transfert coefficient of the analytes, and thus decreases their sampling rates as well, in order to reach a longer field exposure. As for the POCIS, PRC approach was investigated (Camilleri et al., 2012) but not implemented for biotesting achievements (Shaw et al., 2009).

### II. assessment techniques in microalgae laboratory experiments

Ecotoxicology was defined by Truhaut (1977) as “the branch of toxicology concerned with the study of toxic effects, caused by natural and synthetic pollutants, to the constituents of ecosystems, animals (including humans), vegetable, and microbial, in an integrated context.” In ecotoxicology, lethal, and sub-lethal effects can be distinguished. Studies dealing with microalgae in ecotoxicology concern sub-lethal effects, defined as effects occurring at concentrations or doses below those producing direct somatic death (Rand and Petrocelli, 1985). We will present here the two main types of laboratory experiments that are used in order to assess the sub-lethal effects of toxicants on microalgae. These two types of laboratory experiments differ in term of concentrations of toxicant applied and exposure duration. In the first case, the effects of toxicants at high concentrations are assessed during a short time of exposure (typically 96 h) using the dose-response approach. In the second case, microalgae are exposed to concentration of toxicants closed to environmental concentrations for a longer duration (generally more than 1 week).

#### Evaluation of the effects of toxicant at high concentration

##### Dose-response approach

**Figure 1:**
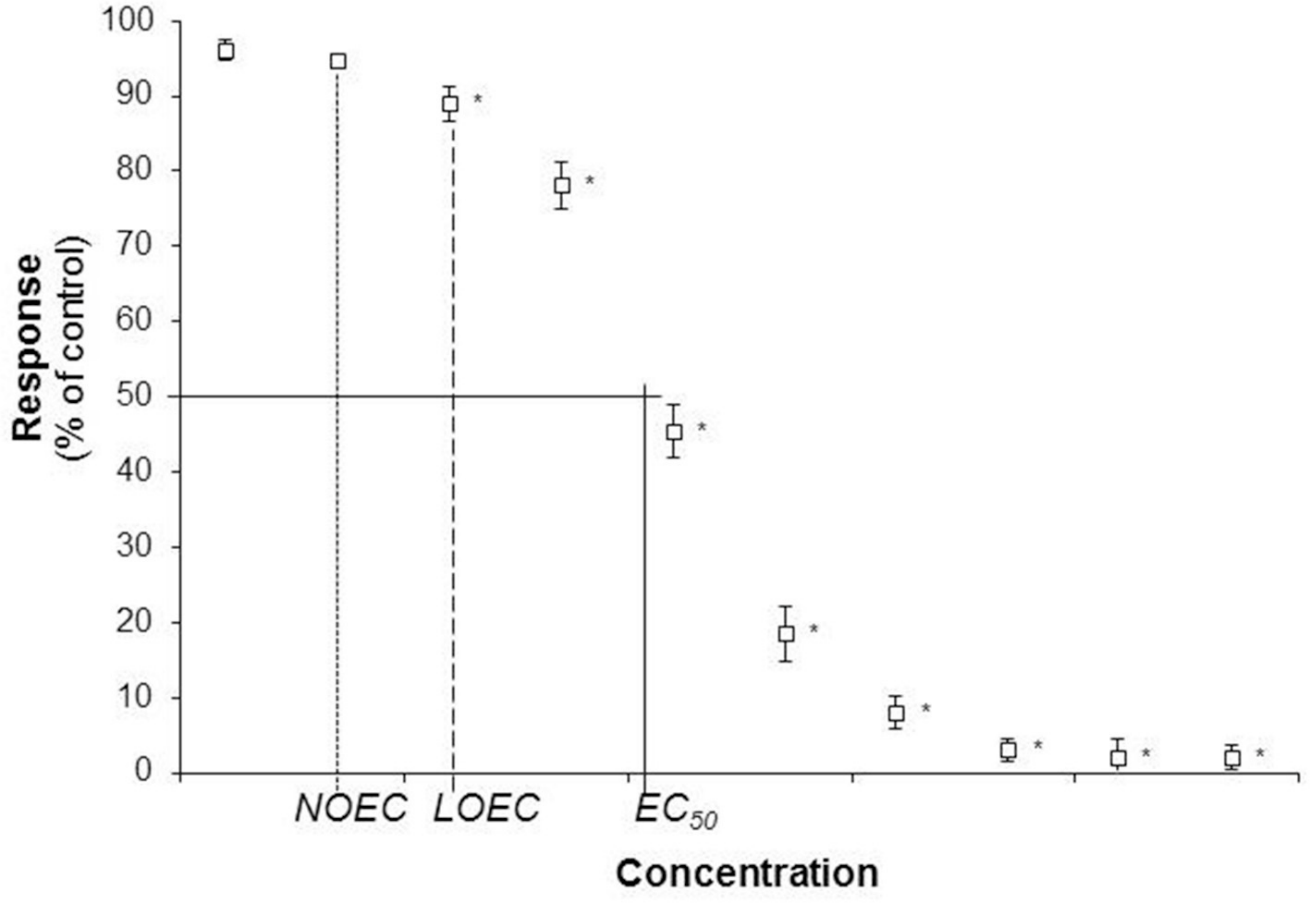
**Illustration of a dose-response curve with the half maximal Effective Concentration (EC_**50**_), the No Observed Effect Concentration (NOEC), and the Lowest Observed Effect Concentration (LOEC)**. Stars illustrate statistical difference from control treatment.

With the dose-response approach (Bruce, 1985, 1987), individuals are exposed to increasing concentrations of toxicant in order to characterize the toxicity of the studied compounds. The endpoint measured to evaluate effect can be any parameter likely to be affected by the toxicant (growth, reproduction, behavior, development etc.). The endpoint measurement for each exposure concentration allowed constructing a sigmoidal dose-response curve (Figure 1). Several methods have been developed to analyse dose-response data (see review in Newman, 2009). Parameters characterizing the toxicity of the studied compound can be extracted from the dose-response curve. The most common been the half maximal Effective Concentration (EC_50_) defined as the concentration inhibiting 50% of the studied parameter compared to the control group. More generally EC_x_ can be calculated (concentration inhibiting X% of the studied parameter compared to the control group). Other parameters like the No Observed Effect Concentration (NOEC) or the Lowest Observed Effect Concentration (LOEC) can be obtained. The NOEC is the highest concentration in a test without significant difference compared to the control and the LOEC is defined as the lowest concentration in a test with a significant effect compared to the control. An important shortcoming of NOEC and LOEC definitions has to be pointed out: NOEC and LOEC values are totally dependent of the exposure concentrations used in the dose-response toxicity testing (Newman, 2009).

##### Dose-response with microalgae as biological model

Dose-response toxicity tests have been used extensively in ecotoxicology in large part because of their ease of operation, rapidity, and low cost. Dose-response toxicity tests with microalgae can be realized at different organization levels from single species to community through simplified assemblages with a limited number of selected species.

The population growth inhibition bioassays on green algae (*Scenedesmus subspicatus* and *Selenastrum capricornutum*) or diatoms (*Skeletonema costatum* and *Phaeodactylum tricornutum*) are normalized bioassays for fresh and marine waters respectively, following the NF EN ISO 8692 (2012) and NF EN ISO 10253 (2006). Other single species toxicity tests have been used with cultures diatoms or green algae isolated from benthic or phytoplankton communities (Leboulanger et al., 2001; Fernández-Alba et al., 2002; Gatidou and Thomaidis, 2007; Magnusson et al., 2008; Roubeix et al., 2011b; Larras et al., 2013; Kim Tiam et al., 2014a). Toxicity tests at the community level generally aimed at characterizing the community tolerance according to the PICT (Pollution Induced Community Tolerance, Blanck et al., 1988) concept (McClellan et al., 2008; Schmitt-Jansen and Altenburger, 2008; Montuelle et al., 2010; Pesce et al., 2010; Rotter et al., 2011; Tlili et al., 2011a,b; Larras et al., 2016).

#### Evaluation of the effects of toxicant at low concentration

Low dose and long term testing are more expensive, longer, and more complicated to realize compared to dose-response testing but offers a better realism regarding environmental exposure. Effect of various toxicant (pesticides, metals, bactericide, PCB) at concentrations closed to environmental contamination levels have been studied; on microcosms or channels systems; on biofilms (Pérès et al., 1996; Gold et al., 2003; Schmitt-Jansen and Altenburger, 2005; Pesce et al., 2006; Morin et al., 2008b, 2010, 2012b; Tlili et al., 2008, 2011c; Debenest et al., 2009; Ricart et al., 2009; Serra and Guasch, 2009; Corcoll et al., 2011; Roubeix et al., 2011a; Bonnineau et al., 2012; Barral-Fraga et al., 2016) and on phytoplanktonic assemblages (Fisher et al., 1974; Gustavson and Wängberg, 1995; Wallen, 1996; Seguin et al., 2002; Echeveste et al., 2014; Pandey et al., 2015). Long-terms effects on phototrophic organisms can be evaluated regarding functional attributes, changes in biomass or effects on the community structure. As explained by Guasch et al. (2016) a big variety of endpoints can be chosen in order to evaluate these effects like photosynthesis and tolerance acquisition (functional attributes), chlorophyll concentration and algal density (changes in biomass), or algal groups, species composition, diatom cell size, teratoforms, and genetic diversity (community structure).

#### The mixture issue: theoretical models and limitations for predicting mixture toxicity

In environmental risk assessment, not taking mixture toxicity into account is likely to lead to hazard underestimation. The possible interactions between substances in combination were first studied in the 80's in pharmacology, with the assessment of human drug interactions (Berenbaum, 1989). Then, the mixture issue was questioned in environmental studies, and ecotoxicological experiments were performed using binary or ternary combinations of toxicants (Roberts et al., 1990; Hickie et al., 1993; Parrott and Sprague, 1993). A large body of literature theoretical models were used to predict the joint effect of mixtures of chemicals based on their individual impacts and specific modes of action. The most commonly used models to predict mixture effects for similar- and dissimilar-acting compounds are, respectively, Concentration Addition (CA, Loewe and Muischneck, 1926) and Independent Action (IA, Bliss, 1939). Both theories assume enhanced effects with increasing numbers of compounds and non-interaction between substances. Therefore, a deviation from the prediction indicates antagonism (weaker effects than predicted) or synergism (stronger effects).

However, these classifications were defined for assessing the impacts of simple mixtures (de Zwart and Posthuma, 2005), and their applicability to more complex mixtures requires to be more systematically tested. The need of characterizing the real impact of complex mixtures toward aquatic organisms, in laboratory experiments, could hardly be achieved using such simplified models, given the multitude of possible cocktails (in terms of composition and concentration), and of direct and indirect targets of the substances. Indeed, the large variety of chemical substances prevents from testing all the combinations likely to be found *in situ*. Additionally, ecotoxicological lack of knowledge on the modes of action of the substances (moreover depending on the target organism, and on the assessment endpoints, e.g., Cedergreen et al., 2007a,b) also limit the application of these models. Therefore, an alternative methodology for mixture toxicity assessment could be the direct use of realistic environmental mixtures.

### III. use of PSD extracts for improvement of mixture toxicity assessment

As passive sampling techniques provide a more complete overview of the real contamination including unknown toxicants, PSD extracts were promising to apprehend the toxicity of environmental mixtures, since they opened perspectives for the use of these “field” extracts as complex contaminants in toxicity testing. PSD extracts present the major advantage that it may be used as a non a priori approach, regarding the composition of the mixture. Besides expected parent substances, PSD extracts may contain breakdown products, as well as any other non-targeted biologically active compounds. Moreover, in ecotoxicity testing, the use of PSD extracts does not require caring about possible interactions between substances (additivity, antagonism, synergism etc.), as the effects of the cocktail of contaminants is measured at a global scale, integrating potential interactions between the accumulated substances (Table 2).

**Table 2:** **Main advantages and limits of the use of polar passive sampler device (PSD) extracts in aquatic ecotoxicology**.

**Use of polar PSD extracts in aquatic ecotoxicology**	
**Advantages**	**Limits**
High degree of representativity relative to environmental contaminations	Questions about the representativeness of the sampled fractions in relation to *in situ* exposure (concentration of compounds may be quite different from *in situ* concentrations due to different sampling rates)
Integration of contamination peaks	No notion of temporal succession of contaminant exposure
Integration of mixtures effects (additivity, synergism, antagonism)	No identification of substances responsible for toxicity
Possibility to use as a black box: Estimation of the global toxicity of the extract without a priori (toxicity of unidentified compounds is integrated)	Only representative of the toxicity of the dissolved phase
	Only sample contaminants in a determined range of polarity depending of the characteristics of the sorbent phase
High ecotoxicological relevance because mimics the uptake of xenobiotics by organisms	Not taking in account the contamination due to the particulate fraction
Case by case study	Difficulty to standardize

#### First approaches using PSD extracts for mixture toxicity testing

Most of the time bioassays using POCIS and Chemcatcher® extracts have been dealing with mixtures present in sewage effluents (Petty et al., 2004; Vermeirssen et al., 2005; Liscio et al., 2009; Balaam et al., 2010) but also with cocktails present in waters subjected to agricultural (Matthiessen et al., 2006) or diverse anthropogenic pressures (Creusot et al., 2010; Jarosova et al., 2012; Bargar et al., 2013; Jálová et al., 2013). PSD extracts were used to determine the toxic potential of different aqueous matrices, in particular to target pharmaceuticals, and more specifically endocrine disruptors. To this end, toxicity was determined using standard tests such as the yeast estrogen screen (YES; Petty et al., 2004; Vermeirssen et al., 2005; Matthiessen et al., 2006; Liscio et al., 2009; Balaam et al., 2010; Bargar et al., 2013) or other reporter gene assays (Creusot et al., 2010; Jarosova et al., 2012; Jálová et al., 2013). This new coupling introduced more environmental realism to ecotoxicological tests, and improved the relative understanding of the effective toxicity of mixtures of pollutants in the aquatic environment.

#### Predicting phytotoxicity of mixture with single species testing

First studies pairing passive sampling with bioassays in order to evaluate the photosynthetic effects of mixtures of contaminants were conducted using bioassays with microalgae as model organisms (Escher et al., 2005; Muller et al., 2007, 2008; Shaw et al., 2009; Vermeirssen et al., 2009, 2010). In these studies, mixture toxicity was evaluated by measuring photosynthetic efficiency after exposure to dilution series of PSD extracts (Table 3). The coupled use of PSD extracts and bioassays on microalgae allowed highlighting the phytotoxicity of the mixtures extracted from waters subjected to agricultural (Muller et al., 2008; Shaw et al., 2009), sewage effluent pressure (Muller et al., 2007; Vermeirssen et al., 2009, 2010), and diverse contaminations (Escher et al., 2006).

**Table 3:** **Studies assessing phytotoxicity of water using PSD extracts and ecotoxicological testing on microalgae**.

**References**	**Study site**	**Landuse**	**Passive sampler**	**Toxicity test (duration)**	**Biological model**
Escher et al., 2006	Vicinity of the Noosa National Park (Australia)	Sewage effluent, urban, domestic	POS (passive sampler for polar organic compounds)	Dose-response (1–25 h)	Single species microalgae (n.a.)
Muller et al., 2007	n.a.	Sewage effluent	POS (passive sampler for polar organic compounds)	Dose-response (n.a.)	Single species microalgae (*Phaeodactylum tricornutum*)
Muller et al., 2008	Brisbane River (Australia)	Agriculture	POS (passive sampler for polar organic compounds)	Dose-response (30 min to 2 h)	Single species microalgae (*Phaeodactylum tricornutum* and *Chlorella vulgaris*)
Shaw et al., 2009	Great Barrier Reef (Australia)	Agriculture	Chemcatcher®	Dose-response (2 h)	Single species microalgae (*Phaeodactylum tricornutum*)
Vermeirssen et al., 2009	Northern part of Switzerland	Sewage effluent	Chemcatcher®	Dose-response (2 h)	Single species microalgae (*Pseudokirchneriella subcapitata*)
Vermeirssen et al., 2010	n.a	Sewage effluent	POCIS	Dose-response (2 h)	Single species microalgae (*Pseudokirchneriella subcapitata*)
Pesce et al., 2011	Ruiné River (France)	Distinct land use	POCIS	Dose-response (2 h)	Biofilms
Morin et al., 2012c	Morcille River (France)	Vineyard	POCIS	Dose-response (48 h) and low dose exposure (14 days)	Biofilms
Booij et al., 2014	The Dutch estuarine and coastal waters	Distinct landuse	POCIS	Dose-response (4.5 h)	Single species microalgae (*Dunaliella tertiolecta*)
Kim Tiam et al., 2014b	Morcille River (France)	Vineyard	POCIS	Dose-response (24 h) and low dose exposure (13 days)	Biofilms
Kim Tiam et al., 2015	Trec River (France)	Growing cereal crops	POCIS	Low dose exposure (14 days)	Biofilms
Foulquier et al., 2015	Morcille River (France)	Vineyard	POCIS	Dose-response (30 min to 3 h)	Biofilms

Study site, landuse, passive sampler, toxicity testing duration and biological model used in the studies are documented. n.a.: non available in the reference.

In these different studies, toxic equivalency concept was used to allow comparing the effect of PSD extracts to a reference compound (i.e., diuron). Diuron equivalent concentrations were calculated for the identified compounds in the extracts; then the toxicity measured in the bioassay and predicted based on chemical analysis results were compared in order to evaluate to which extent extract composition could explain the observed toxicity. In most of the cases, there was a general agreement between the measured and the predicted toxicity. For example, Vermeirssen et al. (2009) showed that six analyzed herbicides inhibitors of PSII correlated very well with toxicity measured in bioassays. Nevertheless, the observed toxicity was not always entirely explained by the chemical analytical results; this could results from the presence of unidentified toxic compounds in the extracts or the occurrence of synergic effects (Shaw et al., 2009; Vermeirssen et al., 2009).

Until now, toxicity testing performed on mixtures of herbicides extracted from different passive samplers have been bioassays on cultures of single microalgae species. Such bioassays have the advantages to be quite cheap and easy to implement but are poor in term of ecological realism. However, proper environmental risk assessment requires higher ecological realism, as pointed out by Artigas et al. (2012). To this end, the toxicity of PSD extracts was recently performed using natural communities likely to be exposed in their environment to these mixtures of chemicals.

#### Application of polar PSD extracts in mixture toxicity assessment using biofilms

**Figure 2:**
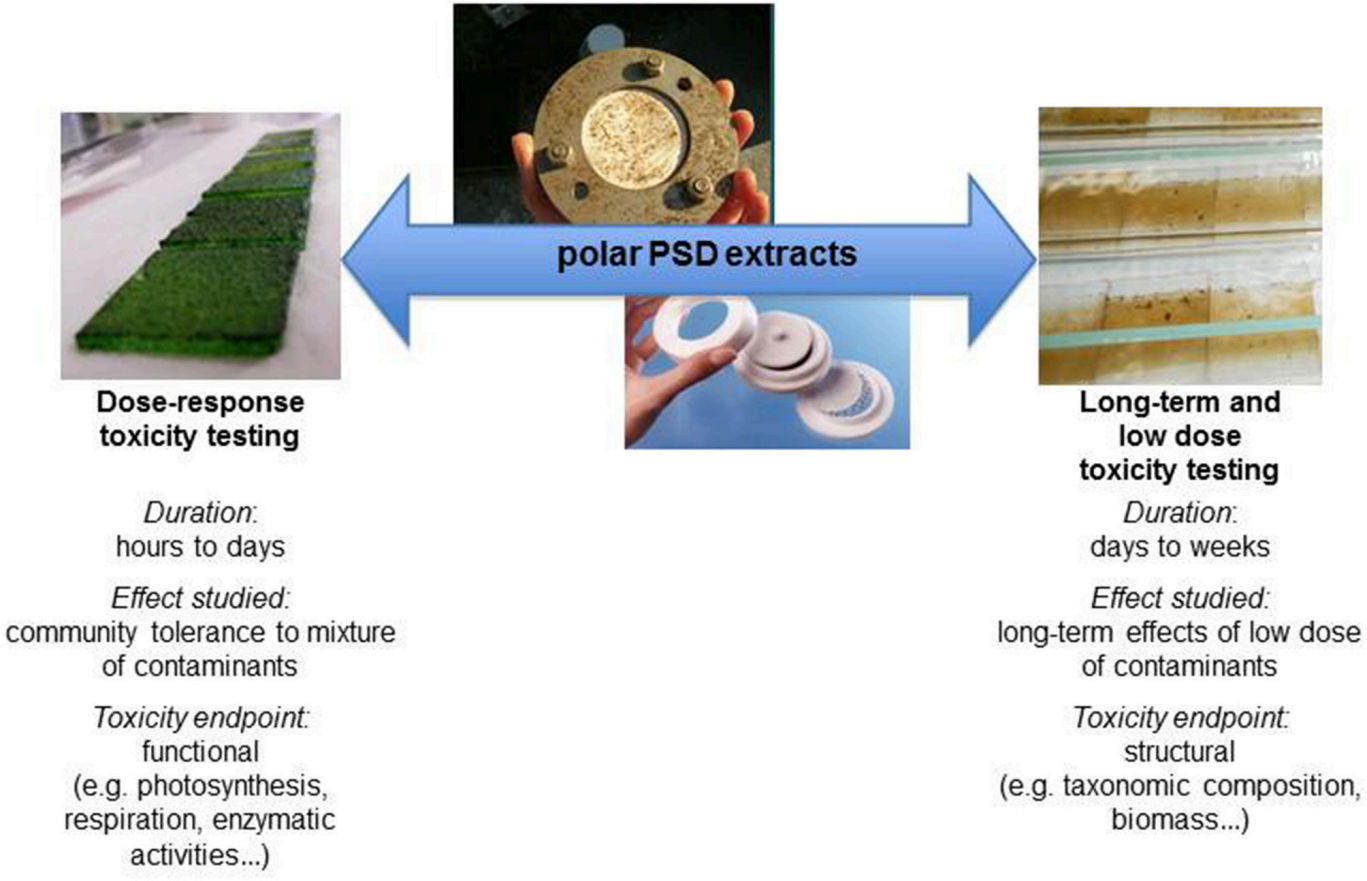
**Duration, effects studied and examples of endpoints used in toxicity testing using polar passive sampler device (PSD) extracts and biofilms**.

In order to increase ecological relevance in environmental risk assessment, it was proposed to realize ecotoxicological testing using PSD extracts on complex communities. In this purpose, two main experimental designs were used. Dose-response curves were realized in order to evaluate community tolerance to mixture. Long-term effects of low dose of contaminants were assessed by long-term exposures (e.g., more than 1 week; Figure 2). In such toxicity testing, community tolerance is generally evaluated with functional parameters (e.g., photosynthesis, respiration, enzymatic activities…) whereas long-term effects of low dose of contaminants can be highlighted studying parameters related to community structure (e.g., taxonomic composition, biomass…).

POCIS extracts were used to assess the impacts of environmental pesticide mixtures with ecologically relevant bioassays with biofilms (Pesce et al., 2011; Morin et al., 2012c; Kim Tiam et al., 2014b). Toxicity testing of pesticide extracted from POCIS was recently performed on biofilms originated from different French rivers subjected to agricultural pressure (Table 4). Biofilms are communities composed by diverse microorganisms (microalgae, bacteria, fungi, etc.) embedded in an exo-polysaccharid (EPS) matrix. They are involved in major processes in water ecosystems functioning, in particular in primary production and organic matter processing. Pesce et al. (2009), besides, evidenced potential role of aquatic microbes in the degradation of pesticide compounds. The biological complexity of biofilms confer them a capacity of response to contaminants that depends on the kind of substances, their concentrations, their modes of actions (that may target, directly or indirectly, one or more of the diverse components of the biofilms), and the duration of exposure. The complementary endpoints available at different scales of biological organization allow to assess distinctly between short-term impacts of contaminants (early visible on functional endpoints) and long-term impairment (visible after long-term exposure on community structure (Sabater et al., 2007). In the same line Escher et al. (2005) proposed the use of a multi-endpoint toolbox to better assess the hazard of toxic compounds, by combining non-specific (or narcotic) and specific endpoints.

**Table 4:** **Toxicity data for biofilms, exposed to increasing concentrations, and low concentration of PSD extracts**.

**Characteristics of study sites**				**Testing**							
		**Contamination level**		**Dose-response curves**				**Low dose and long-term**			
**River of origine, sampling date (Land use)**	**Site**	**Total *in situ* pesticide concentration (ng/L)**	**Major compounds identified (by decreasing importance)**	**Duration**	**Parameter studied**	**Endpoint**	**Environmental concentration factor**	**Duration**	**Exposure level (total pesticides concentration in extract, μg/L)**	**End point studied**	**References**
Ruiné, 2009 (Distinct land use)	Upstream	1200	DEA, DIA, simazine, DET, atrazine	24 and 48 h	Fv/Fm	LOEC	100x	_	_	_	Pesce et al., 2011
	Downstream	1100	DEA, DIA, DET, simazine, atrazine				>100x				
Morcille, 2010 (Vineyard)	Upstream ^*^R	100	Norflurazon, DIA	48 h	Fv/Fm	LOEC	4x	14 days	0.42 to 0.66	Chlorophyll a fluorescence; ΦPSII; FsBl; FsBr; FsGr; DW; AFDM; SIR; diatom cell density; diatom taxonomy	Morin et al., 2012c
	Downstream ^*^C	1700	Dimetomorph, diuron, norflurazon, DCPMU, tebuconazole				11x				
Morcille, 2011 (Vineyard)	Upstream	300	Norflurazon desmethyl, norflurazon	24 h	Fv/Fm	LOEC	5x	13 days	2.16	Fv/Fm; ΦPSII; FsBl; FsBr; FsGr; DW; AFDM; diatom cell density; diatom taxonomy	Kim Tiam et al., 2014b
	Downstream	2300	Norflurazon desmethyl, dimetomorph, tebuconazole, diuron, norflurazon				49x				
Trec, 2012 (Growing cereal crops)	Reference ^*^Ourbise	< d.l.	_	_	_	_	_	14 days	2.83	ΦPSII; FsBl; FsBr; FsGr; DW; AFDM; AEAs; diatom cell density; diatom taxonomy; diatom biovolumes	Kim Tiam et al., 2015
	Contaminated ^*^Trec	1640	Metolachlor OA, metolachlor ESA, metolachlor, acetochlor ESA, acetochlor OA, DEA, acetochlor								
Morcille, 2013 (vineyard)	Upstream ^*^Ref	300	Norflurazon desmethyl, acetochlore, carbendazime	30 min to 3 h	Fv/Fm	LOEC	95x	_	_	_	Foulquier et al., 2015
	Downstream ^*^C*raw*	1000	Norflurazon desmethyl, dimetomorph, tebuconazole, norflurazon, diuron				126x				

Contamination level is detailed in terms of total pesticide concentration and major substances recorded.

DCPMU, 1-(3,4-dichlorophenyl)-3-methyl urea; DEA, desethylated atrazine; DET, deethylterbutylazine; DIA, desisopropyl atrazine; OA, oxanilic acid; ESA, ethanesulfonic acid. ΦpsII: Effective quantum yield of PSII, FsBl, FsBr, and FsGr, fluorescence signals linked to cyanobacteria, diatom and green algae group determination; DW, dry weight; AFDM, ash-free dry mass; SIR, substrate-induced respiration; AEAs, antioxidant enzyme activities.

Environmental concentration factor correspond to concentration factor from which photosynthetic yield was inhibited compared to in situ pesticide concentrations, LOEC corresponds to total pesticides concentration in PSD extract (μg/L) from which photosynthetic yield was inhibited.

* Labeled in the corresponding reference.

Ecotoxicity assessment combining mixture toxicity POCIS extracts (PE) and biofilms as “target organisms” is particularly relevant, given the fact that POCIS accumulate pesticides, in particular herbicides (Table 1), and that biofilms are principally composed of autotrophic organisms. Biological toxicity testing requires large amounts of contaminants. POCIS where shown to accumulate sufficient amount of contaminants to run such tests (e.g., 287 mL/day for acetochlor ESA for 600 mg of sorbent, Fauvelle et al., 2014).

##### Community tolerance to mixture

Risk assessment of mixtures has mainly been performed through dose-response bioassays. In particular, PE were applied at increasing mixture concentrations to perform PICT approaches. The PICT principle relies on the hypothesis that when a community is exposed to a toxicant the abundance of sensitive organisms to this toxicant will decrease in favor of less sensitive organisms. This shift in community structure is leading to a higher tolerance of the new formed community compared to the previous one. In such approach, community tolerance is generally assessed with dose-response curves. The works based on the PICT concept, using PE considered as a “blackbox” of contaminants, allowed to highlight previous exposure of the biofilms, or not, to these cocktails. The ecological significance of combined PICT and PE approaches proved to be relevant, for biofilms sampled from rivers presenting different contamination profiles in term of concentrations and/or nature of compounds detected (Pesce et al., 2011; Morin et al., 2012c; Kim Tiam et al., 2014b; Foulquier et al., 2015). The PICT responses of field biofilms were compared in three study sites subjected to distinct kinds of agricultural pressure (increasing vineyard occupation from upstream to downstream in Morcille river, distinct land use along the Ruiné river and growing cereal crops at the Trec river; Table 4). Dose-response curves performed with PE sampled at the downstream (Ruiné and Morcille) or contaminated (Trec) site allowed to evidence differences in the sensitivity to PE mixtures depending on the origin of the biofilms. Apart from Foulquier et al. (2015), significant higher tolerance was measured for biofilms originated from the most contaminated site (noted contaminated for Trec river and downstream for Morcille river in Table 4). These results confirmed the relevance of using PE as “blackbox” complex contaminants to reveal biofilm exposure history. Notably, the mixtures were generally dominated by breakdown products. The ecotoxicity of pesticides metabolites is less studied than that of their parent compound, although they may be much more toxic (Sinclair and Boxall, 2003). In particular, norflurazon desmethyl was shown to inhibit biofilm photosynthesis at concentrations much lower than norflurazon (Kim Tiam et al., 2014a).

With the combined PICT and PE approaches, previous biofilm pesticide exposure can be evidenced by the measure of biofilm tolerance to the pesticide mixture. Tolerance (measured in term of ECx, LOEC…) can thus be easily compared for biofilms originated from different sites. However, in these approaches it is difficult to compare results from different studies.

##### Impacts of low dose and long-term exposure

With the success of PE use for dose-response bioassays on biofilms, new perspectives were opened for further applications, including their use as composite contaminants for low dose and long-term exposure in laboratory experiments. The use of diluted PE in long-term exposure experiments is particularly relevant to empirically demonstrate the potential toxicity of environmental cocktails, as they occur in rivers. Demonstrating the toxicity, or not, of PE at low (environmentally realistic) dose is required for a renewed risk assessment and the revision of current quality standards taking mixtures into account (as previously recommended by Chèvre et al., 2006).

First attempts provided evidence that PE could be used for those purposes (Morin et al., 2012c), but required experimental improvements. Fluvial biofilm was exposed in artificial channels to low dose mixtures of contaminants during 2-week periods. In Kim Tiam et al. (2014b, 2015), changes in biofilm biomass, growth, taxonomic structure and function (algal fluorescence and detoxification related endpoints) observed over 2 weeks confirmed the potential of the use of PE in long-term exposures to assess the impacts of low dose mixtures, as found in the field. These impacts differed depending on biofilm history (previously exposed or not, and thus species composition and adaptation Kim Tiam et al., 2014b). These works highlighted that long-term exposure to low dose mixtures impacts aquatic organisms at a relatively short time scale. They showed the relevance of POCIS extracts in such laboratory approaches.

### IV.questioning and future challenges

The uncertainties related to the use of PSD extracts for toxicity testing on microalgae cover issues related to both, passive sampling techniques (Table 2) and assessment techniques in microalgae laboratory experiments.

#### How to consider variation in sampling rates of the compounds trapped by PSD?

Most of the uncertainties related to the use of PSD extracts for toxicity testing are preoccupations shared with environmental chemists (Harman et al., 2012; Morin et al., 2012a). They notably question the representativeness of the sampled fractions in relation to *in situ* exposure. In the case of POCIS, a wide range of compounds is accumulated and a posteriori calculation of time-weighted average concentrations is possible for substances whose sampling rates have been calibrated (Table 1). Therefore, when the sampling rates are well-defined, environmental concentration can be either expressed as equivalent of the EC_50_ (Kim Tiam et al., 2014b) estimated from dose-response curves or simulated, under controlled laboratory conditions, with appropriate dilution levels of the PSD extracts (Kim Tiam et al., 2015). However, with the aim of testing the mixture toxicity by using POCIS extracts, the composition of the extract may be quite different from *in situ* concentrations. This issue is mainly due to the variability of the sampling rates (e.g., POCIS sampling rates typically ranging from 80 to 300 mL/day, depending on the compound properties and flow velocity; Harman et al., 2012). To overcome this limitation, several approach can be used such as *in situ* calibration (Mazzella et al., 2010; Ibrahim et al., 2013), use of passive flow monitor (PFM; O'Brien et al., 2011) or appropriate PRC (Mazzella et al., 2010; Dalton et al., 2014; Lissalde et al., 2014), or just checking sufficient flow velocity (e.g., ≥2 cm/s) on the field (Di Carro et al., 2014).

#### Are PSDs able to cover the entire range of contaminants present in the water phase?

PSD extracts do not reveal the entire complexity of the studied system, since each sampler have a defined selectivity in terms of polarity for organic compounds, or charge for inorganic species (Table 1). One passive sampler could only integrate substances with similar characteristics, and could thus avoid interactions between species having opposite physico-chemical properties. In the case where rivers are subjected to contaminations by chemical families having very different physico-chemical characteristics, using only one PSD to calculate water chemical concentrations can lead to underestimation (in term of number of chemicals) of the contamination. In this case, the use of different PSDs can be needed in order to better represent the mixture of chemicals the aquatic organisms are exposed to. Extracts of these different PSD could thus be used together (weighted by their average sampling rates) to perform toxicity assessment in order to better evaluate mixture effects. This is particularly relevant in sites impacted by vineyard treatments where organic pesticides (i.e., diuron, norflurazon, dimetomorph, tebuconazole, norflurazon desmethyl…) and inorganic pesticides (i.e., copper and arsenic) are used together (Morin et al., 2012c). This could permit to better take in account additivity, synergism, or antagonism effects in environmental risk assessment of mixtures. For example, several studies demonstrated that the co-occurrence of copper and glyphosate reduces their respective toxicity (Tsui et al., 2005; Zhou et al., 2013).

#### How are peaks of contamination integrated by PSD?

Studying the risk linked to time-varying exposure using mixtures require PSD to adequately sample pulses, especially in the case of substances with a rapid onset of action. A lagtime in accumulation of some compounds was described in POCIS, due to mass transfert through the water boundary layer and the membrane of the device (Belles et al., 2014; Lissalde et al., 2014). Regarding polar PSD like POCIS and Chemcatcher®, some study showed their applicability for integrating peaks of contamination (at least 10 times the background concentration) for 24–96 h periods, and for moderately polar pesticides (log K_ow_ = 1.79–3.21; Mazzella et al., 2008; Shaw and Mueller, 2009). However, integration of very short event (i.e., few hours only) and for a large range of pesticides must be addressed, since depending on the lagtime of the chemical of interest, POCIS could be unsuitable to capture short peak events, which could notably affect aquatic life.

#### Is the fraction sampled by PSD representative of the compounds available for aquatic organisms?

Harman et al. (2012) put forward that passive samplers mimic the uptake of xenobiotics by organisms, thus making the use of PSD extracts more relevant ecotoxicologically. Dissolved waterborne compounds, i.e., the fraction sampled by polar PSDs, are generally expected to be the most bioavailable to organisms and thus responsible of the toxicity. However, there are processes other than passive diffusion that are expected to occur in organisms: active uptake, metabolization, etc. Moreover, in the case of biofilms, contaminated particles can be entrapped by the EPS matrix, resulting in close contact between particulate toxicants and organisms. This was demonstrated for metals, the particulate fraction being the most correlated with bioaccumulation (Morin et al., 2008a). There is, thus, an urgent need to determine the toxicokinetics of pesticides in biofilms, and whether bioaccumulated quantities are, or not, a better estimate to assess toxicity (Sappington et al., 2011). In this complex matrix where internal physicochemistry differs from water characteristics, the toxicant may be released and made available to the organisms. The particulate exposure pathway can thus not be ruled out, especially in the case of hydrophobic pesticides (Coat et al., 2011). On the contrary Poulier et al. (2014) showed that polar pesticides are in majority found in the dissolve fraction.

#### Can we identify the compound(s) responsible for toxicity in mixtures?

The global toxicity of pesticide mixtures can thus be assessed in a non a priori approach based on the use of a “blackbox” contaminant. Kim Tiam et al. (2015) demonstrated that long-term and low dose exposure to POCIS extracts and to a reconstituted mixture of the 12 major pesticides drove similar functional and structural changes in biofilm. In that study, the adverse effects of PSD extracts were thus due to a limited number of toxic compounds.

Petrovic et al. (2011) showed a very limited number of toxic compounds often explain the adverse effects of complex environmental mixtures. In such cases, the identification of the compound(s) responsible for toxicity can be expected from EDA (Effect-Directed Analysis, Brack, 2003), which combines chemical and biological analytical approaches. Booij et al. (2014) used this approach in order to identified the main chemical stressors that negatively affect photosynthetic efficiency in marine microalgae of the Dutch estuarine and coastal waters. However, important drawbacks of these approaches rely on the fact they are laborious and time consuming, especially when diverse bioassays (endpoints) are required to appropriately assess potential toxicity. Moreover technical bias can not allow to identify the compound(s) responsible for toxicity in case of lose of toxicity of the entire extract reconstituted from the fractions. From an analytical point of view, the development of non-targeted techniques such as the use of time-of-flight high resolution mass spectrometry could help identifying the major contaminants of the PSDs “blackbox” (Hug et al., 2014; Guibal et al., 2015).

#### How to take into consideration intra and inter-species sensitivity variations in single species testing?

Enormous databases (e.g., ECOTOXicology knowledgebase, Pesticide Properties DataBase, etc.) containing toxicity testing data have been built over decades. Dose-response testing based on a variety of algal species represents the large majority of toxicity testing. The EC_50_, NOEC, and LOEC are the most widely used parameters for toxicity characterization and comparison. Different species (Leboulanger et al., 2001; Larras et al., 2012) or clones (Ivorra et al., 2002; Roubeix et al., 2012) can have significant differences regarding to their sensitivity to toxicant. Because of that comparing and interpreting EC_50_, NOEC, and LOEC from different studies can be challenging. One way to easily compare toxicity data could be the wide use of toxicity reference compound and toxic units approach as it is commonly applied when assessing PSD extract toxicity in particular in Effect-Directed Analysis studies (Shaw et al., 2009; Vermeirssen et al., 2009).

#### Do we need more consistency between toxicity studies on microalgae?

In normalized toxicity tests using microalgae, experimental conditions regarding media and reagents, equipment, procedure, validity criteria, and calculations are set in order to standardize the assay (NF EN ISO 8692 10253, 2006; 2012). Non-normalized toxicity testing is also widely used in order to better taking in account species diversity and environmental conditions. In these studies, algal concentration, exposure medium, or exposure duration vary and often make comparison difficult between studies as shown by Eklund and Kautsky (2003) in a review on toxicity testing with marine macroalgae. In this study, the authors pointed out the need for method standardization. This underlines the need for a document stipulating the minimum information for publication of dose-response experiments.

This need for more consistency between toxicity studies has been recently pointed out in the case of studies applying the PICT concept (Lambert et al., 2015; Tlili et al., 2015). Tlili et al. (2015) propose practical guidance and identified a list of research issues that should be considered in order to make the PICT approach an ecologically relevant risk assessment tool of chemicals in aquatic systems. One major issues concerned standardization of PICT measurements regarding control of microbial colonization, use of a standardized medium for tolerance measurements, and normalization of tolerance values.

#### Can we deal with fluctuating concentrations and time-dependent effects in bioassays?

Intermittent discharges of pesticides in agricultural watersheds are generally linked to rain events occurring after crop application (e.g., Rabiet et al., 2010). They result, for aquatic organisms, in fluctuating exposure alternating peaks and lower concentrations. In laboratory experiments, nominal concentrations are generally defined for each exposure condition. These concentrations are wanted to be maintained constant through the entire test duration. In reality, toxicant concentrations often differ from the nominal ones due to adsorption to the experimental units, volatilization, or degradation. Nevertheless, these variations of concentration can be minimizing by using flow-through test compared to static-renewal test or static toxicity test. Almost all toxicity testing are based on constant concentrations of exposure what is poorly representative of aquatic organisms' exposure in the environment and result in several shortcomings. The impacts of pulse insecticide exposure scenarios on fish and invertebrates have been widely studied (see review in Chèvre and Vallotton, 2013). In contrast, the responses of algae to intermittent herbicide contamination have mainly been assessed through laboratory bioassays with the model microalgae *Scenedesmus vacuolatus* (Vallotton et al., 2008a,b, 2009). Copin et al. (2015) modeled the effects of sequential exposures to isoproturon on the growth of *Scenedesmus vacuolatus*, taking into account pulse duration and concentration. Highest inhibition was caused either by short-term high dose or by long-term low dose scenarios. Fluctuating exposure was also shown to impact biofilms, depending on the number of pulses (Tlili et al., 2008, 2011c) and on peak duration (Gustavson et al., 2003; Laviale et al., 2011).

## Conclusions

Classical approaches in mixture toxicity consist in using models incremented with data obtained from single compound toxicity tests, these approaches permit to apprehend the relations of additivity, antagonism or synergism occurring in the mixture but are restricted to mixtures containing a very limited number of substances which are poorly representative of environmental contamination. The capacity of PSDs to accumulate diverse contaminants permits to better estimate the environmental contamination. Their accumulation properties also made PSDs excellent tools for mixture toxicity assessment by the direct use of their extracts in ecotoxicological testing. The main advantages of such approach is that mixture effects are integrated, metabolites are taking in account, and it is a without a priori approach (PSD extracts are used as “blackbox” composite contaminant).

In this paper we presented studies where polar PSDs and toxicity assays at different biological scales have been successfully used for pesticide mixture toxicity assessment. The coupled use of POCIS or Chemcatcher® extracts and toxicity testing on microalgae single species has demonstrated its relevance for predicting phytotoxicity of mixtures as shown by the general agreement between the toxicity measured in the bioassay and predicted based on chemical analytical results. In order to increase ecological relevance biofilms have been used as biological model to evaluate POCIS extracts toxicity. This approach has been successfully used for rivers subjected to agricultural or distinct landuse pressure resulting in a contamination dominated by herbicides. It permitted to evidence acquisition of tolerance and long-term changes in community structure, demonstrating that PSD extracts can successfully be used for this purpose. Nevertheless, coupling PSDs and toxicity assays also presents some limitations that require being considered. The future studies will have to focus on questions concerning, sampling rates, sampling range, integration of peaks of contamination by PSD, bioavailability, identification of compounds responsible for toxicity, intra and inter-species sensitivity variations, consistency between toxicity studies and time-dependent effects in bioassays.

## Author contributions

SK: first author, main contributor on the coupling of passive sampling and algae part. VF: second author, main contributor on the passive sampler part. SM: third author, second contributor on the coupling of passive sampling and algae part. NM: last author, second contributor on the passive sampler part.

### Conflict of interest statement

The authors declare that the research was conducted in the absence of any commercial or financial relationships that could be construed as a potential conflict of interest.
